# The Role of Kynurenine and Its Metabolites in Comorbid Chronic Pain and Depression

**DOI:** 10.3390/metabo12100950

**Published:** 2022-10-06

**Authors:** Onella Athnaiel, Charmaine Ong, Nebojsa Nick Knezevic

**Affiliations:** 1 Advocate Illinois Masonic Medical Center, Department of Anesthesiology, Chicago, IL 60657, USA; 2 Chicago Medical School, Rosalind Franklin University of Medicine and Science, North Chicago, IL 60064 USA; 3 Department of Anesthesiology, University of Illinois, Chicago, IL 60612, USA; 4 Department of Surgery, University of Illinois, Chicago, IL 60612, USA

**Keywords:** depression, chronic pain, kynurenine pathway, quinolinic acid, kynurenic acid, inflammation, psychiatric disorders, tryptophan metabolism, comorbidity

## Abstract

Chronic pain and depression affect millions of people worldwide, and their comorbidity tends to exacerbate the severity of each individual condition. Intersecting brain regions and molecular pathways could probably explain the unique yet complex bidirectional relationship between these two disorders. Recent studies have found that inflammatory reactions, frequently identified in both chronic pain and depression, stimulate certain enzymes in the kynurenine pathway, while concurrently suppressing others. Kynurenine, a major tryptophan derivative, and its metabolites have been implicated in several inflammation-associated pain syndromes and depressive mood disorders. Due to inflammation, 95% of tryptophan is metabolized via the kynurenine pathway, which drives the reaction towards the production of metabolites that have distinct roles in the pathophysiology of these disorders. Diminished levels of the neuroprotective metabolite, kynurenic acid (KYNA), and elevated levels of the neurotoxic metabolite, quinolinic acid (QUIN), have been frequently identified in human patients formally diagnosed with these disorders, as well as animal models commonly used in medical research. This review not only explores the epidemiology of comorbid chronic pain and depression, but also highlights the involvement of kynurenine and its metabolites, specifically KYNA and QUIN, in these pervasive conditions.

## 1. Introduction

Chronic pain is defined as persistent or intermittent pain that lasts for more than three months [1], and is associated with a diminished quality of life, increased healthcare costs, opioid dependence, reduced productivity, and a higher chance of developing psychiatric disorders such as depression, anxiety, and substance abuse disorders [2]. It is among the most common chronic conditions in the United States; in 2019, the National Health Interview Survey revealed that at least 50.2 million American adults (20.5%) reported pain on most days or every day [3]. Each year more than 500 billion dollars are spent on healthcare costs related to pain management and opioid dependence [4]. This exceeds the amount spent on cancer, diabetes, and heart disease combined [2].

## 2. Epidemiology of Comorbid Chronic Pain and Depression

Clinical studies have revealed bidirectional relationships between chronic pain and depression; pain is associated with the persistence of depression, and depression is associated with persistent pain. This bidirectional relationship can potentially be explained by the major molecular pathways that transpire in both conditions, which will be explored throughout this article.

A 2019 study by Orhurhu et al. analyzed the National Inpatient Sample (NIS) dataset from 2011 to 2015 and used the ninth and tenth revisions of the International Classification of Diseases (ICD-9 & ICD-10) to identify 9.3 million patients with a diagnosis of chronic pain. Out of these 9.3 million patients, 2.2 million (22.9%) were diagnosed with comorbid depression. The majority of these 2.2 million patients were female (67.5%) and the mean age was 59, with the highest proportion of patients with comorbid depression falling between 45 and 64 years of age. Additionally, patients in zip codes within the lowest income quartile had the highest proportion of comorbid depression (31%), while those in zip codes within the highest quartile had the lowest proportion of depression (16.8%) [5]. These trends are supported by other epidemiological studies showing an increased prevalence of depression among women [6,7] and low-income adults [8,9] in particular, and emphasize the complexity of the relationship between chronic pain and depression.

Several studies have revealed an increased prevalence of pain among patients with depression, and vice versa. Rayner et al. studied 1204 chronic pain patients attending an initial consultation at a specialized pain treatment center, and found that 60.8% (732/1204; 95% CI 58.0–63.6) met the criteria for mild, moderate, or severe major depressive disorder (MDD) [10] as assessed by the Patient Health Questionnaire-9 (PHQ-9), a nine-item survey for screening, diagnosing, monitoring, and measuring the severity of depression [11]. This is in stark contrast to the World Health Organization’s estimation that only 5% of adults around the world suffer from the disorder [12]. Another study of 3566 patients attending outpatient psychiatric facilities found that 59.1% of patients with a depressive disorder reported pain [13]. There was also a significant correlation between the intensity of pain (assessed via scores on the visual analog scale) and the intensity of depression (evaluated by the 17-item Hamilton depression scale). Additionally, the duration of the current depressive episode was longer in patients reporting pain, suggesting that depression can influence a patient’s pain experience independent of a nociceptive stimulus [13]. On the other hand, Rayner et al. found that pain patients with comorbid depression also reported a greater extent of pain (assessed by the number of regions affected) and were more likely to report experiencing generalized pain (90.6% among depressed patients, vs. 83.2% among nondepressed patients, *p* < 0.001) [10]. They also reported a significantly greater pain interference—the extent to which pain interferes with physical, mental, and social activities [14]—than those without depression (*p* < 0.001) [10]. These findings highlight the bidirectional nature of the relationship between these two conditions.

Other studies have also identified associations between severity of pain and severity of depressive or anxiety symptoms. De Heer et al. analyzed data from the Netherlands Study of Depression and Anxiety (NESDA), a longitudinal study of 2981 patients recruited from the community, primary care providers, and specialized mental health care facilities [15]. Each participant was interviewed by a trained researcher using the World Health Organization’s DSM-IV-based composite international diagnostic interview (CIDI), a reliable and valid assessment for depressive and anxiety disorders [16]. Of the sample, 396 had a current diagnosis of depression, 543 had a current diagnosis of anxiety, 762 had a current diagnosis of both depression and anxiety, and 628 had a remitted disorder. A total of 652 participants had no psychopathology. Severity of depressive symptoms was assessed with the quick inventory of depressive symptomatology-self-report (QIDS-SR), a shortened version of the inventory of depressive symptomatology. Severity of anxiety symptoms was assessed with the Beck anxiety inventory (BAI). Pain was not evaluated in either assessment. Pain levels were then assessed using the seven-item chronic pain grade (CPG) scale, an instrument commonly used to grade pain based on intensity and pain-related disability. CPG scores can be used to calculate five grades of chronic pain, ranging from grade zero (no pain symptoms) to grade four (high disability and severely limiting pain, regardless of intensity) [15].

Analysis of the results showed that having a depressive or anxiety disorder increased the patient’s odds of experiencing highly disabling and severely limiting pain (CPG 4). A total of 80% of patients with CPG four pain also had a current diagnosis of depression (14.3%), anxiety (13.5%), or both (52.1%). Of the 170 patients (5.7%) reporting no pain symptoms (CPG 0), 48% had no psychopathology and 19.4% had a remitted disorder. Those with comorbid depressive and anxiety disorders were least likely to report no pain symptoms. Among those with pain, the highest odds ratios were seen in comorbid depression and anxiety [15]. These findings are supported by other studies identifying an increased co-occurrence of chronic pain with depression and cardiovascular disease [17]. Additionally, increased severity of depressive and anxiety symptoms was significantly associated with highly disabling and severely limiting pain. See Table 1 for a summary of these findings.

## 3. Neural Mechanisms Linking Chronic Pain and Depression

The complex relationship between chronic pain and depression and anxiety has been thought to be at least partially related to the pathophysiological pathways they share. Recent studies have found considerable overlap in the regions of the brain involved in mood management and the sensing of injury and body pains, including the prefrontal cortex (PFC), insular cortex, anterior cingulate, amygdala, hippocampus, and thalamus [18]. Decreased volumes of the PFC [19,20], hippocampus [21], and thalamus [22] have been observed in patients with depression and were found to also be closely related to the severity of the disorder [22]. Postmortem studies have also revealed that individuals with depression have a reduction in PFC neuronal body size, suggesting a decrease in synaptic activity [23]. This is supported by microarray gene profiling revealing reduced expression of synapse-related genes and loss of excitatory synapses in both the PFC and hippocampus of individuals with MDD [19]. Meanwhile, other reports have identified a link between the PFC and pain development via the nucleus accumbens [18]. One study found a correlation between increased activity in the medial PFC and the intensity of chronic back pain [24]. These findings suggest that the co-occurrence and development of depression and pain may be associated with similar changes in neuroplasticity. 

Tissues can be damaged in many ways, namely infection, trauma, oxygen deprivation, chemicals, and heat; each of these pathways leads to inflammation. Once inflammation is initiated, a cytokine cascade follows. There are many molecular mechanisms that take place downstream of this pathway, which ends in the brain [25]. Interactions between neural circuits in the brain and inflammatory pathways can lead to behavioral responses and may contribute to the development of depression at the molecular level [26]. Neuroinflammation is an innate immune response of the nervous system to infection, injury, or neurodegeneration [27]. Furthermore, chronic inflammation can lead to changes in neuronal structures, transitioning sickness to depression and acute pain to chronic pain. Inflammatory signals have been associated with changes in neurotransmitter metabolism and neuroplasticity by affecting depression-related areas of the brain via the blood–brain barrier. Even in healthy individuals, inflammatory stimuli, such as vaccination and/or lipopolysaccharide (LPS) injections, were shown to have a negative impact on mood and cognition. These findings were also associated with increased plasma levels of inflammatory cytokines tumor necrosis factor-alpha (TNF-α) and interleukins six and 1Ra [25].

**Figure 1:**
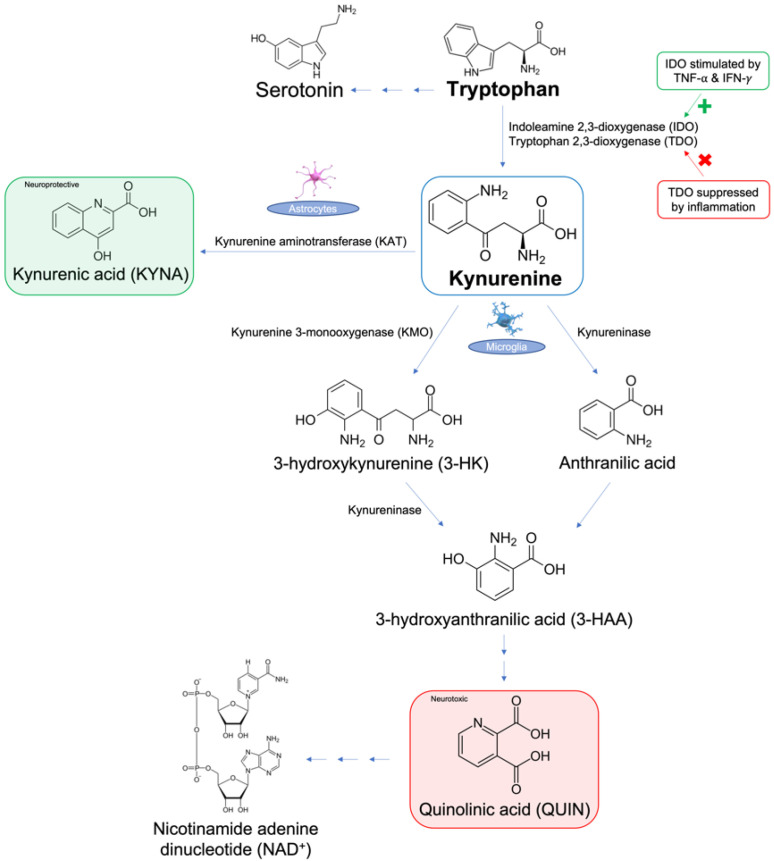
Overview of the metabolism of tryptophan via the kynurenine pathway.

The kynurenine pathway in particular has been a point of interest due to its involvement in both chronic pain and psychiatric disorders. Kynurenine is a metabolite of the aromatic ring amino acid, tryptophan (Trp), and is produced in the pathway synthesizing nicotinamide adenine dinucleotide (NAD^+^) from ingested Trp, illustrated in Figure 1 [28]. Trp is converted to kynurenine via two main enzymes: indoleamine 2,3-dioxygenase (IDO) and tryptophan 2,3-dioxygenase (TDO) [29]. During inflammation, TDO is suppressed [30], while IDO is activated, mainly by interferon-gamma (IFN-γ) and TNF-α [25]. Kynurenine is further metabolized via three other pathways: (1) synthesis of 3-hydroxykynurenine (3-HK) and its subsequent metabolites 3-hydroxyanthranilic acid (3-HAA) and quinolinic acid (QUIN) by kynurenine 3-monooxygenase (KMO) in microglia, (2) synthesis of kynurenic acid (KYNA) by kynurenine aminotransferase (KAT) II in astrocytes or skeletal muscle in periphery, and (3) synthesis of anthranilic acid by kynureninase in microglia [30]. Kynurenine is neuroactive and capable of exerting neurotoxic effects in certain regions of the brain, such as the hippocampus and PFC [31]. It regulates the activity of glutamatergic N−methyl−d−aspartate (NMDA) receptors, allowing them to induce toxicity by generating reactive species. In addition, kynurenine can cause deficiencies in the pathway of Trp-linked NAD^+^ synthesis. Several of kynurenine’s metabolites also have neurotoxic and neuroprotective effects in response to inflammation, which play a key role in both chronic pain and depression [31]. For instance, QUIN is a potent NMDA receptor agonist, which can indirectly induce excitotoxicity, mitochondrial damage, oxidative stress, destabilization of the cellular cytoskeleton, and disruption of autophagy, as a result of stimulating other intracellular pathways that propagate these negative outcomes [32]. This article will explore the involvement of the kynurenine pathway and its major metabolites in the co-occurrence of depression and chronic pain [31].

The kynurenine pathway is the main route of tryptophan metabolism, producing the end-product nicotinamide adenine dinucleotide (NAD^+^). In this pathway, indoleamine 2,3-dioxygenase (IDO) metabolizes tryptophan to kynurenine and is upregulated by pro-inflammatory cytokines, such as tumor necrosis factor-alpha (TNF-α) and interferon-gamma (IFN-γ). Kynurenine is further metabolized to (1) neuroprotective kynurenic acid (KYNA) via kynurenine aminotransferase (KAT), (2) 3-hydroxykynurenine (3-HK) via kynurenine-3-monooxygenase (KMO), or (3) anthranilic acid via kynureninase. Further metabolism occurs to 3-HK by kynureninase, producing neurotoxic quinolinic acid (QUIN). Recent studies have implicated decreased levels of KYNA [30,31] and increased levels of QUIN [30,31] in the pathogenesis of both chronic pain and depression. The rest of this article will focus on the role of kynurenine in chronic pain and depression in the context of clinical and animal studies.

## 4. Kynurenine Pathway and Chronic Pain

Previous clinical studies have identified an association between excessive IDO levels and inflammation-induced pain. As previously mentioned, IDO is the main enzyme in the kynurenine pathway that converts Trp to kynurenine and other active metabolites. Staats Pires et al. found that IDO levels and kynurenine/Trp ratios were significantly higher in patients with back pain compared to healthy controls [32]. A different study of 113 female patients conducted by Barjandi et al. (2019) included 17 with temporomandibular disorders myalgia, 40 with fibromyalgia, and 56 healthy controls. Venous blood was drawn and kynurenine and Trp ELISA tests were run on all participants. The results showed a significant negative correlation between Trp plasma levels and pain intensity (*p* < 0.001), suggesting that low Trp levels are associated with higher pain intensity. However, there was a significant positive correlation between the kynurenine/Trp ratio and pain intensity (*p* < 0.001), suggesting that higher kynurenine levels are associated with increased pain. This confirms that most Trp is metabolized in the kynurenine pathway in cases of inflammation-induced pain [33].

However, Trp is also needed to produce the neurotransmitter serotonin [29,30,31]. As such, IDO and serotonin levels are inversely related. In an inflammatory state, continuous consumption of Trp via IDO and the subsequent kynurenine pathway leads to abnormally low levels of serotonin. This is consistent with the low serotonin/Trp ratio and elevated kynurenine/Trp ratio seen in chronic arthritis inflammatory pain model rats [32].

## 5. Kynurenine Pathway and Depression

Not only is the kynurenine pathway involved in chronic pain, but it has also been found to be involved in psychiatric disorders. A meta-analysis conducted by Ogyu et al. revealed that: (1) both KYNA and kynurenine levels were decreased in patients with depression compared with healthy controls, (2) ratios of both KYNA/QUIN and KYNA/3-HK were lower in patients with depression compared with healthy controls, and (3) there were no differences in QUIN and 3-HK levels between the two groups. These findings suggest that the kynurenine pathway plays a vital role in depression. Ample evidence suggests there is increased inflammation in patients with depression, and the reduced KYNA/3-HK ratio in patients with depression further confirms this, suggesting that the KMO pathway is activated as a result of depression [30]. Additionally, Barjandi et al. identified a significant negative correlation between anxiety symptoms, which were evaluated using the hospital anxiety and depression scale (HADS) and the general anxiety disorder scale (GAD-7), and kynurenine/Trp ratios in patients with fibromyalgia [33]. This finding further confirms the connection between low kynurenine levels and increased psychiatric symptoms.

QUIN is also considered to be a major contributor to depression by acting on the hippocampus. As one of the limbic structures involved in mood and behavior, the hippocampus cannot synthesize QUIN; however, it possesses NMDA receptor 2A and 2B subtypes, which have a very high affinity for QUIN. Consequently, QUIN can have neurotoxic effects on the hippocampus and promote depression. This neurotoxicity can be displayed as energy dysfunction, oxidative stress, transcription factors, cytoskeletal disruption, behavioral changes, and cell death [34]. Another meta-analysis investigating 22 studies and 1894 unmedicated patients with depression found decreased levels of kynurenine and KYNA, but elevated levels of QUIN, further confirming the neurotoxic effects of this key product of the kynurenine pathway [34].

## 6. Kynurenine Metabolites in Chronic Pain and Depression

There is an ample amount of research exploring chronic pain and depression comorbidity in patients, with the aim of understanding the pathophysiology of both conditions and possibly explaining the reason for this highly prevalent comorbidity. In a retrospective observational study conducted by Pope et al., 298 chronic pain patients were given the patient reported outcomes measurement information system (PROMIS) survey, followed by a urine test prior to beginning therapy. The survey assessed many domains, such as fatigue, pain interference, physical function, sleep disturbance, anxiety, depression, and ability to participate in social roles and responsibilities. Pain intensity was measured with the numeric rating scale (NRS) which was also included in the survey. The urine test was used to investigate pain biomarkers and their relationship with the domains assessed in the survey. The results showed that this population had worse pain than the general population, and they displayed a tendency toward depression, anxiety, fatigue and sleep disturbances. Additionally, 87.2% of all patients had at least one abnormal biomarker in their urine. The most notable of all biomarkers was QUIN, one of the key metabolites of the kynurenine pathway. KYNA was also elevated in 33% of these patients and was strongly associated with pain interference (*p* = 0.015) [35].

Groven et al. studied 48 patients with chronic fatigue syndrome, 58 fibromyalgia patients, and 54 healthy controls, and they examined kynurenine and its metabolites in their blood plasma. When compared with healthy controls, patients with chronic fatigue syndrome had lower KYNA/QUIN ratios, and patients with fibromyalgia had lower ratios of KYNA/3-HAA. This finding suggests that their symptoms could be attributed to a lack of neuroprotective metabolites [36]. Other studies have found that treatment with IFN-α results in increased depressive symptoms and eventual progression to MDD, potentially due to elevated kynurenine/Trp levels [25]. Another study by Erhardt et al. (2013) assessed the amount of KYNA and QUIN in the cerebrospinal fluid (CSF) of 64 patients who had attempted suicide and were medication free and compared them to 36 healthy controls. Results showed that QUIN, but not KYNA, was elevated in the CSF of the patients that had attempted suicide. QUIN levels in those patients were approximately 300% of those in healthy patients [37].

Involvement of the kynurenine pathway and its metabolites has also been identified in patients with schizophrenia, another psychiatric disorder. Schizophrenia is often associated with depression and anxiety and has also been found to be associated with decreased pain sensitivity, although 27% of patients report somatizations that closely correlate with emotional distress. Patients with schizophrenia also display different sensory perceptions, manifesting as insensitivity to hot or cold-induced pain. Depression and anxiety in these patients are closely related to somatic symptoms, such as pains, aches, and flu-like malaise. Many of these findings are attributed to low-grade inflammation and IDO pathway activation. One study recruited 84 patients with schizophrenia and 40 healthy controls, diagnosing schizophrenia according to DSM-IV-TR criteria [38]. Interviews were conducted to assess fatigue, somatic symptoms, and severity of depression and anxiety. The fibromyalgia and chronic fatigue syndrome rating (FF) scale was used to assess fatigue, including symptoms such as: muscle pain, muscular tension, fatigue, concentration difficulties, failing memory, irritability, sadness, sleep disturbances, autonomic disturbances, irritable bowel, headache, and a flu-like malaise. The Hamilton depression rating scale (HAM-D) was used to assess depression, and the Hamilton anxiety rating scale (HAMA) was used to assess anxiety. Both scales contained items similar to the ones on the FF scale. Results showed that 53.75% of patients with schizophrenia had somatic symptoms, the most common ones (in descending order) being: concentration difficulties, failing memory, muscle pain, irritable bowel, and irritability. After considering the depression and anxiety scores, negative symptoms appeared to be marginally significant, suggesting that physical symptoms, depression, and anxiety form a group of symptoms that are tightly connected and highly associated with schizophrenia. Molecular studies testing immunoglobulin responses showed high IgM activity against QUIN in these patients, suggesting its contribution to the comorbidity of physical and psychiatric symptoms. Decreased KYNA levels were also found, suggesting a lack of neuroprotective molecules [38]. Although schizophrenia is a different psychiatric disorder, the presence of anxiety, depression, and physical symptoms in conjunction with the kynurenine pathway metabolites further confirms the involvement and significance of this pathway in the development of these symptoms, as it is especially seen in inflammatory episodes.

However, brain tissues from 45 postmortem patients with depression that were found to have reduced inflammation, marked by low levels of TNF-α and IFN-γ, demonstrated less activity in the kynurenine pathway. These findings suggest that inflammation plays a key role in activating this pathway; the presence of depression without inflammation is not sufficient to upregulate kynurenine metabolism. In addition, the majority of kynurenine activity was noted in the ventrolateral prefrontal cortex (VLPFC) [39,40]. Future studies may investigate the kynurenine pathway in other regions of the brain in postmortem patients with depression. See Table 2 for a summary of the clinical findings.

## 7. Kynurenine Metabolites in Animal Studies

Preclinical studies have also shown consistent results in animal models of depression, despite the lack of clarity on how representative animals can be when it comes to depression and anxiety. Upon inducing inflammation with LPS, cytokine production was increased, and IDO mRNA was seen in microglia. In addition to these molecular findings, LPS administration also elicited depressive-like behaviors in animal models after inducing acute sickness, which later disappeared, while depression persisted. C57BL/6J Mice infected with Mycobacterium bovis, Bacillus Calmette-Guérin (BCG) displayed depressive-like symptoms and increased Trp catabolism via the kynurenine pathway for an extended period of time [41]. A similar study included additional pretreatment with antidepressants and found that depressive-like symptoms could be avoided, despite the inflammation caused by LPS and cytokines. This suggests that inflammation is an important factor contributing to or even possibly causing depression as seen in humans [25].

Older literature has shown that intracerebroventricular injections of LPS and TNF-α are sufficient to produce depressive-like symptoms in rats. Hippocampal injections of TNF-α in a stereotaxic surgery on rats were deemed to be sufficient to increase thermal hypersensitivity for three weeks post-surgery and allodynia between 12–21 days after surgery. However, administering cytokine antagonists was found to be effective at combating these symptoms [25]. In one study conducted on Wistar rats, injections of KMO inhibitor, which blocks neuronal glutamate receptors by kynurenate, alleviated both nerve injury-induced depressive-like symptoms and mechanical allodynia [42]. This protective element was most likely due to control of the leukocyte infiltration in the brain [43]. Inhibiting KMO resulted in kynurenine being converted to KYNA instead, and therefore also reduced the amount of QUIN, the toxic metabolite of the kynurenine pathway that is typically elevated in inflammatory states [44]. In addition to reducing depressive-like symptoms, KMO inhibition seemed to be effective at reducing pain receptors as well. These findings suggest that inhibition of one of the enzymes of the kynurenine pathway can be protective against the undesirable outcomes of depression and chronic pain.

Other studies have found that inhibiting IDO or IFN-γ can be effective at protecting against depression. Presence of the IDO enzyme is essential to induce depression-like symptoms. As previously mentioned, IFN-γ is one of the main activators of IDO. In mice that are IFN-γ deficient (IFNγR mice), IDO is not activated, and thus the mice are protected against depressive-like symptoms, despite the induction of an inflammatory response upon inoculation with BCG [39,45]. 

Zhang et al. investigated the effect of ketamine injections on pain-induced depression in SD rats. The rats were injected with Freund’s adjuvant to elicit allodynia, depression, and hyperalgesia, which were later confirmed by mobility and sucrose preference tests. Fourteen days later, one group of rats was injected with 1 mL ketamine (20 mg/kg), while the other group was injected with 1 mL saline as a vehicle treatment. Results showed that one injection of ketamine was effective at reducing allodynia and immobility time and increasing sucrose preference. These findings suggest that ketamine has an effect on treating the comorbidity of inflammatory pain and consequent depression. In this same study, hippocampal levels of IDO, kynurenine, and Trp were evaluated upon the administration of ketamine. Results showed that both IDO and the kynurenine/Trp ratio were decreased due to the ketamine injection, suggesting that pain-relieving and antidepressant effects were achieved via the kynurenine pathway [46]. 

Zhou et al. also conducted a study to investigate the effect of IDO on depression and comorbid pain. C57/BL6J and *Ido1−/−* mice were included in the study, and surgery was performed to obtain spared nerve injury as a model for neuropathic pain. The surgery resulted in decreased social interaction and increased immobility time, confirming depression-like behavior in mice. Although results showed that this induction of inflammation did not lead to an increase in IDO in the brain, there was a significant increase in the liver. Consequently, higher kynurenine levels and kynurenine/Trp ratios were only observed in the plasma, and not in the hippocampus or the frontal cortex, providing interesting findings compared to the study that did find significant increases in hippocampal tissues. Mice that did not have IDO expression did not show a decrease in social interaction and an increase in immobility following the spared nerve injury, suggesting that this enzyme is necessary for the expression of this behavior. However, the test showed that they still developed allodynia, showing that the lack of IDO is not enough to prevent pain [47].

## 8. Conclusions

Millions around the globe suffer from comorbid chronic pain and depression. Elucidating the molecular pathways involved in both of these conditions is critical in developing effective treatments and improving patient outcomes. Upon exploring the literature on chronic pain and depression, inflammation was found to be a crucial factor in both morbidities. Further investigation of inflammation pointed to the kynurenine pathway, one of the dominant sequela of Trp, an essential amino acid. The kynurenine pathway is extensive, producing many metabolites that have different reactions in various regions of the body and brain.

As highlighted throughout the article, kynurenine levels and kynurenine/Trp ratios were consistently elevated in patients with depression and chronic pain, and they were also noticeably abnormal in animal studies. Regarding the metabolites of kynurenine, KYNA was deemed to be neuroprotective, and was regularly found at lower levels in those same patient populations. On the other hand, QUIN was found to be neurotoxic, especially to the hippocampus, and was abnormally elevated in the plasma of patients with depression and chronic pain. However, it was not only the metabolites that played a role in these morbidities. The IDO enzyme also elevated kynurenine levels, which likely led to certain metabolites, notably QUIN, being produced in excess, while others, such as KYNA, were suppressed. IDO was found to be stimulated by inflammation, demonstrating another link between the kynurenine pathway and the pathogenesis of these morbidities. Overall, a pattern was observed in all studies involving depression and chronic patients: elevated QUIN levels and decreased KYNA levels. Future studies might consider investigating the role these specific metabolites can play in the treatment of these common morbidities.

## Figures and Tables

**Table 1:** Summary of studies analyzing the frequency of comorbid chronic pain and depression.

Study	Patient Population	Patient Demographics	Assessments/Diagnosis Methods	Patients with Comorbid Disorder
Orhurhu et al., 2019 [5] PMID 31561661	9.3 million inpatients with ICD-9/10 diagnosis of chronic pain	Mean age: 59 67.5% female	ICD-9, ICD-10 for both chronic pain and depression	2.2 million patients (22.9%) with comorbid depression
Rayner et al., 2016 [10] PMID 26963849	1204 chronic pain patients referred to a specialty outpatient pain management unit	Median age: 47 67% female	Depression (diagnosis and severity) assessed using the PHQ-9	407 patients (33.8%) with comorbid severe MDD 256 (21.3%) with comorbid moderate MDD 69 (5.7%) with comorbid mild MDD
Aguera-Ortiz et al., 2011 [13] PMID 21055826	3566 patients attending outpatient psychiatric facilities	Mean age: 49.4 71.3% female	Depression diagnosed based on the DSM-IV-TR, intensity assessed by the 17-item Hamilton depression scale Pain assessed by survey questions, pain intensity assessed by VAS	2107 patients (59.1%) with depression also reported pain
de Heer et al., 2014 [15] PMID 25330004	2981 patients recruited from the community, primary care providers, and specialized mental health care facilities	Mean age: 41.9 66.4% female	Depression/anxiety diagnosed via DSM-IV-based CIDI, severity of depression assessed via QIDS-SR, severity of anxiety assessed via BAI Pain assessed using the 7-item CPG Scale	396 (13.3%) with current diagnosis of depression, 543 (18.2%) with current diagnosis of anxiety, 762 (25.6%) with current diagnosis of both depression and anxiety, 628 (21.1%) with remitted disorder 80% of patients with CPG 4 pain also had a current diagnosis of depression (14.3%), anxiety (13.5%), or both (52.1%)

Abbreviations: International Classification of Diseases, ninth and tenth revision (ICD-9 and ICD-10), Patient Health Questionnaire-9 (PHQ-9), major depressive disorder (MDD), *Diagnostic and Statistical Manual of Mental Disorders 4th Edition Text Revision* (DSM-IV-TR), visual analog scale (VAS), composite international diagnostic interview (CIDI), quick inventory of depressive symptomatology-self-report (QIDS-SR), Beck anxiety inventory (BAI), chronic pain grade (CPG).

**Table 2:** Summary of clinical studies investigating the kynurenine pathway metabolites in chronic pain and depression patients.

Study	Morbidity	Sample Type/Test	Number of Patients	Main Findings
Barjandi et al., 2019 [33] PMID: 31545529	Fibromyalgia and temporomandibular disorders myalgia	Venous blood tests and ELISA tests	113 total: 17 temporomandibular disorders myalgia; 40 fibromyalgia; 56 healthy controls	Negative correlation between Trp ratio and pain intensity Positive correlation between kynurenine/Trp ratio and pain intensity
Groven et al., 2021 [36] PMID: 34090138	Chronic fatigue syndrome and fibromyalgia	Venous blood tests	160 total: 48 chronic fatigue syndrome; 58 fibromyalgia; 54 healthy controls	Fibromyalgia: low KYNA Chronic fatigue syndrome: low KYNA/QUIN ratio Confirming the neurotoxicity and neuroprotective aspects of QUIN and KYNA, respectively
Erhardt et al., 2013 [37] PMID: 23299933	Depression and suicide attempt survivors	Cerebrospinal fluid tests	100 total: 64 suicide attempt survivors; 36 healthy controls	Low KYNA levels Elevated QUIN levels, at 300% of healthy controls Confirming the neurotoxicity and neuroprotective aspects of QUIN and KYNA, respectively
Brown et al., 2021 [39] PMID: 34029552	Depression	Postmortem brain tissues	45	Low TNF-α and IFN-γ Low kynurenine activity suggesting the necessity of inflammation to observe kynurenine elevation in depression
Pope et al., 2021 [35] PMID: 34512007	Depression, anxiety, and fatigue	Urine tests	298	Depression patients had worse pain scores than the general population 87.2% had abnormal QUIN and KYNA levels
Kanchanatawan et al., 2017 [38] PMID: 28258445	Schizophrenia	Plasma molecular studies	124 total: 84 schizophrenia; 40 healthy controls	53.75% had somatic symptoms (muscle pain) Depression and anxiety tightly connected to physical symptoms High activity against QUIN Low activity against KYNA Consistent with the pattern observed in other studies

Abbreviations: Enzyme-linked immunosorbent assay (ELISA), interferon-gamma (IFN-γ), kynurenic acid (KYNA), quinolinic acid (QUIN), tryptophan (Trp), tumor necrosis factor-alpha (TNF-α).
